# Chaperone Hsp70 (HSPA1) Is Involved in the Molecular Mechanisms of Sleep Cycle Integration

**DOI:** 10.3390/ijms23084464

**Published:** 2022-04-18

**Authors:** Valentina V. Simonova, Mikhail A. Guzeev, Irina V. Ekimova, Yuri F. Pastukhov

**Affiliations:** Sechenov Institute of Evolutionary Physiology and Biochemistry, Russian Academy of Sciences, 44 Thorez pr., 194223 St. Petersburg, Russia; miguz85@mail.ru (M.A.G.); pastukh36@mail.ru (Y.F.P.)

**Keywords:** *Hspa1* expression, deep sleep, REM sleep, sleep cycles, sleep deprivation, NRPO

## Abstract

The molecular mechanisms of sleep cycle integration at the beginning and the end of the inactive period are not clear. Sleep cycles with a predominance of deep slow-wave sleep (SWS) seem to be associated with accelerated protein synthesis in the brain. The inducible Hsp70 chaperone corrects protein conformational changes and has protective properties. This research explores (1) whether the *Hspa1* gene encoding Hsp70 protein activates during the daily rapid-eye-movement sleep (REMS) maximum, and (2) whether a lower daily deep SWS maximum affects the *Hspa1* expression level during the subsequent REMS. Combining polysomnography in male Wistar rats, RT-qPCR, and Western blotting, we reveal a three-fold *Hspa1* upregulation in the nucleus reticularis pontis oralis, which regulates REMS. *Hspa1* expression increases during the daily REMS maximum, 5–7 h after the natural peak of deep SWS. Using short-term selective REMS deprivation, we demonstrate that REMS rebound after deprivation exceeds the natural daily maximum, but it is not accompanied by *Hspa1* upregulation. The results suggest that a high proportion of deep SWS, usually observed after sleep onset, is a necessary condition for *Hspa1* upregulation during subsequent REMS. The data obtained can inform the understanding of the molecular mechanisms integrating SWS and REMS and key biological function(s) of sleep.

## 1. Introduction

In humans, most other mammals and birds, sleep is traditionally divided into two distinct states, slow-wave sleep (SWS) and rapid-eye-movement sleep (REMS) [1]. SWS is generally associated with slow-wave activity (SWA), i.e., low-frequency high-amplitude waves on electroencephalogram (EEG). Deep SWS, or “delta-sleep” with 0.5–4 Hz SWA, prevails right after sleep onset, whereas REMS increases towards the end of the inactive period. In adult mammals, SWS predominates the daily sleep cycle, leaving only 10% to 50% of total sleep time to REMS, depending on the species [1]. The prevalence of SWS within the sleep cycle can be explained by numerous physiological functions associated with this state, such as decreased metabolism, reduced secretion of stress-related hormones, lowered body and brain temperature, suppressed viscerosomatic functions (e.g., muscle tone, gastrointestinal motility, etc.), as well as increased production of growth hormone, testosterone, prolactin, insulin, and glycogen [2].

SWS is often termed non-REM sleep in contrast to “active” REM sleep, a state that was first reported in humans by Nathaniel Kleitman and Eugene Aserinsky in 1953 [3,4]. Michel Jouvet soon discovered that the activated electroencephalogram (EEG) during REMS in cats is associated with a flat electromyogram indicating a complete muscle atonia, and hence introduced the term “paradoxical sleep” [5,6]. Paradoxical sleep, or REMS, is now identified by high-frequency low-amplitude waves on EEG (i.e., cortical EEG desynchronization), rapid eye movements, phasic movements of the head and body coupled with overall muscle atonia, unregular breathing and heartbeat, and vivid dreams [4,7,8,9]. Despite almost 70 years of research, many sleep researchers point out that evolutionary origins, key biological functions, and molecular mechanisms governing REMS and its relations with SWS within ultradian and daily sleep cycles remain unclear [2,10,11,12,13]. Foundational questions such as whether REMS “is a thing” [11] and which molecular mechanism underlies the SWS-REMS cycle [14] persist to this day.

Among hypotheses concerning key functions of sleep [10], the restorative function attracts attention. It relates to the ATP “surge” and the increase in available energy recourses during deep SWS, allowing for the intensification of biosynthesis [15,16,17], including protein synthesis acceleration in the brain [18,19]. Nakanishi et al. have prominently demonstrated that deep SWS, unlike light SWS, contributes to accelerated protein synthesis: the intensity of protein synthesis in 35 brain regions positively correlates with the amount of deep SWS in adult macaques [18]. This restorative function of sleep is hard to overestimate since the biosynthesis of proteins and other macromolecules promotes neuronal recovery, supports cellular proteostasis, and thus underlies the normal functioning of the central nervous system. However, it is unknown whether REMS and its temporal relations with SWS are involved in the restorative function of sleep.

Intensified biosynthesis during SWS can be accompanied by the accumulation of misfolded proteins [20], which threaten neuronal survival and may lead to neurodegeneration. HSP70 chaperones (70 kDa Heat Shock Proteins) prevent the accumulation of misfolded proteins [21], and sleep/rest deprivation induces the expression of stress-inducible chaperones in various brain areas [22,23,24]. Prolonged wakefulness leads to the accumulation of unfolded and misfolded proteins that provoke an unfolded protein response and trigger chaperone expression, along with the suppression of other proteins expressed in the brain [25]. Notably, inducible Hsp70 (gene *Hspa1*) and 78 kDa glucose-regulated protein Grp78 (gene *Hspa5*) chaperones are expressed independently from a hormonal stress reaction to sleep deprivation [26].

It is hypothesised [2] that REMS provides similar conditions adequate for the expression of inducible chaperones within the sleep–wake cycle. REMS is characterised by an overall organism activation (except for the muscular system): blood flow intensifies, brain temperature increases, and phasic movements, ponto-geniculo-occipital waves, and rapid eye movements are identified. Therefore, REMS in a way resembles wakefulness, which induces chaperone expression [27], and may be viewed as an endogenous stressor [28,29]. Increased Hsp70 expression could occur during the REMS-dominated cycles that precede awakening and are several hours away from sleep onset when the deepest SWS is observed. In turn, Hsp70 can target the misfolded proteins that might accumulate while the protein synthesis rate grows during deep SWS [18,19,20], which precedes REMS-enriched cycles and prevails right after the sleep onset (i.e., at the beginning of the night in humans or in the morning in rats and other nocturnal animals).

This study focuses on the inducible Hsp70 chaperone since it plays a key role in protein folding and protection against conformational changes. It is one of the ubiquitous proteins and a central “coordinator” of proteostasis in mammalian cells. Hsp70 maintains all stages of protein folding and refolding, as well as facilitates disaggregation of unfolded proteins and the degradation of irreversibly damaged molecules in 26S proteasome [21,30,31,32]. Although Hsp70 functions in neural tissue under physiological conditions, its amount can increase dramatically in response to the accumulation of unfolded or damaged proteins, which emphasises the important role of the Hsp70 chaperone in supporting cellular proteostasis. Chaperone complexes consisting of Hsp70 and Hsp110 can act as “protein disaggregase” and convert protein aggregates into the soluble form [33]. Other members of the HSP70 family, including Hsc70 and Grp78, do not possess this property. 

Inducible Hsp70 is shown to be involved in the regulation of the sleep–wake cycle as well. Hsp70 promotes SWS [34], which is mediated through its influence on γ-aminobutyric acid (GABA) and adenosine neurotransmission in the ventrolateral preoptic area (vlPOA) of the hypothalamus [35,36]. Hsp70 knockdown within the vlPOA reduces sleep and increases wakefulness [37]. These data indicate that Hsp70 contributes to the mechanisms of SWS modulation and the realisation of its restorative function. 

To the best of our knowledge, previous research on sleep and chaperones (e.g., [16,23,24,27,38,39]) covered neither the changes in Hsp70 expression during REMS nor the molecular mechanisms governing interrelation between this function of REMS and deep SWS in preceding sleep cycles. In the present study, we aimed to identify whether the expression of the *Hspa1* gene, encoding inducible Hsp70 chaperone, is enhanced during REMS in rats and whether *Hspa1* expression depends on the preceding natural daily maximum of deep SWS.

## 2. Results

### 2.1. Natural REMS Is Accompanied by Hspa1 Upregulation

**Figure 1:**
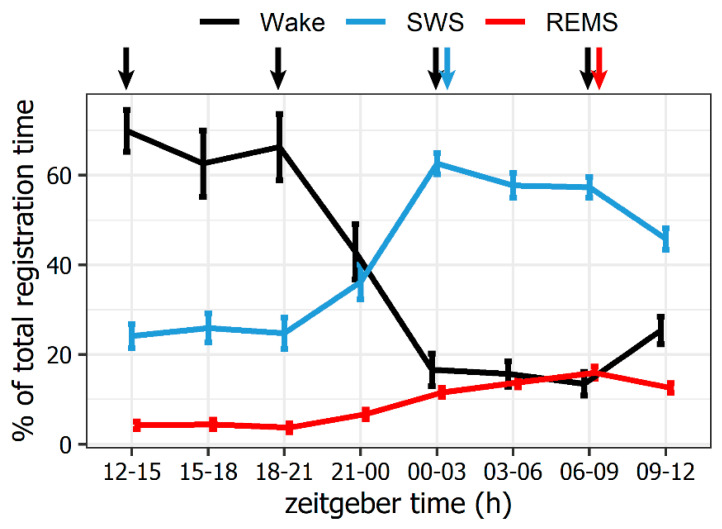
Total time of vigilance states comprising the natural daily sleep–wake cycle in Wistar rats (*n* = 10). The natural daily maximum of slow-wave sleep (SWS) is registered after the sleep onset at ZT00-03, while REM sleep (REMS) peaks at the end of the inactive (lights-on) period at ZT06-09. Y-axis: % of total registration time. X-axis: zeitgeber time, hours. Arrows indicate the time points when 6 groups of rats (*n* = 6 in each group) were sacrificed at daily maximums of wakefulness (ZT12-15; 18-21), SWS (ZT00-03) and REMS (ZT06-09) for the subsequent real-time qPCR analysis of the brain tissue. Colours signify the preceding vigilance state: black—wakefulness, blue—slow-wave sleep, red—REM sleep. Values are shown as mean ± s.e.m, *n* = 10 rats.

The polysomnographic study demonstrated the natural circadian dynamics of SWS and REMS in rats (*n* = 10). SWS dominates in the daily sleep–wake cycle during the first 3 h of the lights-on period, reaching its maximum at ZT01-02 (36 ± 1.0 min per hour); median SWS episode duration also peaks at this time point at 3.1 ± 0.98 min. The daily maximum of the deep SWS stage coincides with the SWS peak. The total time (TT) of SWS over the ZT00-03 interval is 2.5 times higher compared to the mean SWS TT registered during the inactive (lights-off) period (Figure 1). The daily maximum REMS is registered during the second half of the lights-on period at ZT06-09 (9.6 ± 0.8 min per hour); the median REMS episode duration reaches 2.4 ± 0.70 min. REMS TT here is 4.3 times higher than the mean REMS TT registered during the lights-off period (Figure 1). The daily minimum REMS (2.2 ± 0.6 min per hour), with the median REMS episode duration of 1.8 ± 0.47 min, is registered in the ZT18-21 interval.

Relying on these data, rats (*n* = 6 in each group) were sacrificed at ZT01-02 (during SWS) and ZT07-08 (during REMS). *Hspa1* expression levels at these time points were compared against values measured during wakefulness at ZT01-02 or ZT07-08, as well as against wakefulness during the lights-off period when SWS and REMS are at their daily minimums (the average value of ZT13-14 and ZT19-20; Figure 1). 

**Figure 2:**
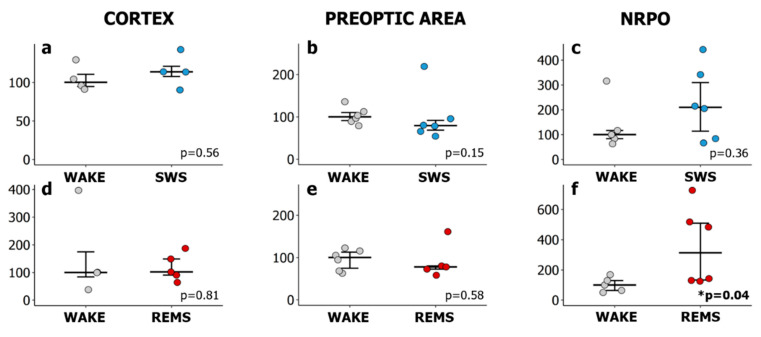
*Hspa1* expression increases in the nucleus reticularis pontis oralis during the natural daily maximum of REM sleep in the lights-on period. Relative *Hspa1* expression in (**a**,**d**) the sensorimotor cortex (CORTEX), (**b**,**e**) the preoptic area of the hypothalamus (PREOPTIC AREA) and (**c**,**f**) the nucleus reticularis pontis oralis (NRPO) in slow-wave sleep (SWS, upper row) or REM sleep (REMS, lower row). The values were calculated using the 2^−ΔΔCt^ method, where the reference group was in wakefulness either at ZT01-02 (for SWS) or at ZT07-08 (for REMS). WAKE (grey)—rats were sacrificed after a 10 min episode of wakefulness at ZT01-02 or ZT07-08, SWS (blue)—after 5–5.5 min episode of slow-wave sleep at ZT01-02, REMS (red)—after 2.5–3.5 min episode of REM sleep at ZT07-08. Values are shown as median ± 75/25 percentile. Each dot represents an independent sample (animal). *p* values were calculated using the Mann–Whitney U test with a corresponding “WAKE” group as a control.

Real-time qPCR analysis revealed no significant changes in *Hspa1* expression in the sensorimotor cortex and the POA between sleep states and wakefulness when comparing either with the lights-off period (Kruskal–Wallis test, H(2, N = 17) = 1.72, *p* = 0.4; H(2, N = 21) = 0.48, *p* = 0.8) or with the corresponding wakefulness group (Figure 2a,b,d,e). 

In NRPO, SWS does not affect the level of Hsp70 mRNA (Figure 2c). No significant changes of *Hspa1* expression were identified in SWS or REMS compared to wakefulness in the lights-off period (Kruskal–Wallis test: H(2, N = 24) = 1.25, *p* = 0.5). Notably, *Hspa1* expression was upregulated at the natural daily peak of REMS. During the second half of the lights-on period (ZT07-08), REMS was accompanied by a three-fold increase in *Hspa1* expression in the NRPO compared to the wakefulness at the same timepoint (Figure 2f).

**Figure 3:**
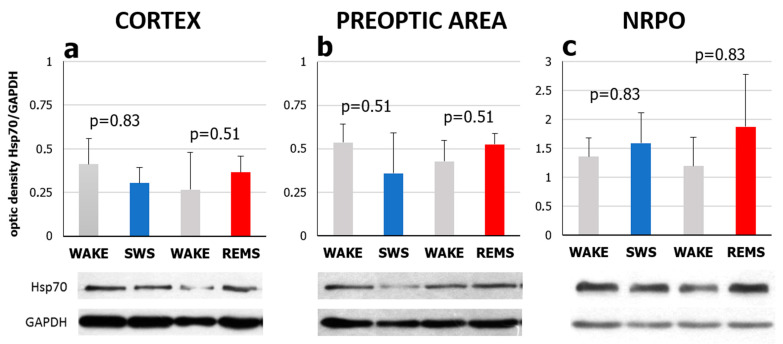
Hsp70 protein levels in (**a**) the sensorimotor cortex, (**b**) the preoptic area of the hypothalamus and (**c**) nucleus reticularis pontis oralis (NRPO) during slow-wave sleep (SWS) and REM sleep (REMS). Western blot analysis of the brain structures was run with the antibodies against inducible Hsp70. Hsp70 protein level is presented in relative units of optical density against the level of house-keeping protein GAPDH. Data from rats sacrificed after 5–5.5 min of SWS at ZT01-02 or after 2.5–3.5 min of REMS at ZT07-08 were compared with the data from rats after 10 min of wake at the same time points. X-axis—vigilance states: WAKE—rats were sacrificed after an episode of wakefulness, SWS—after slow-wave sleep, REMS—after REM sleep. Values are shown as mean ± s.e.m, *n* = 3 for each group. *p* values were calculated using the Mann–Whitney U test with a corresponding “WAKE” group as a control. Representative immunoblots are shown under the respective graphs.

To determine whether the natural daily sleep–wake cycle affects Hsp70 protein levels in the rat brain, immunoblotting with antibodies against inducible Hsp70 was used. No significant changes in Hsp70 chaperone level were identified in the sensorimotor cortex, the POA, or the NRPO between the lights-on and lights-off period (data not shown), nor between vigilance states (Kruskal–Wallis test: H(2, N = 9) =3.82, *p* = 0.15; H(2, N = 9) = 0.27, *p* = 0.88; H(2, N = 9) = 1.689, *p* = 0.43, according to the brain area; Figure 3).

**Figure 4:**
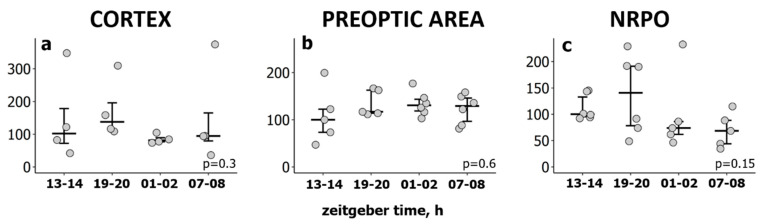
Circadian rhythm has no significant effect on *Hspa1* expression within the studied rat brain areas during wakefulness. Relative *Hspa1* expression level in (**a**) the sensorimotor cortex, (**b**) the preoptic area of the hypothalamus and (**c**) the nucleus reticularis pontis oralis (NRPO) during wakefulness over the day. To access *Hspa1* expression across 24 h, 4 groups of rats were decapitated after 10 min episode of wakefulness at: ZT13-14, ZT19-20, ZT01-02, ZT07-09. The values were calculated using the 2^−ΔΔCt^ method, where the reference group was wakefulness at the beginning of the lights-off (inactive) period (ZT13-14). Each dot represents an independent sample (animal). *p* values were calculated using Kruskal–Wallis H test.

When comparing the *Hspa1* expression in the sensorimotor cortex, the POA or the NRPO during wakefulness across the 24 h period, no significant differences were identified (Kruskal–Wallis test: H(3, N = 16) = 3.79, *p* = 0.3; H(3, N = 22) = 1.74, *p* = 0.6; H(3, N = 22) = 5.38, *p* = 0.15, according to the brain area, Figure 4). These data suggest that the circadian rhythm has no significant effect on *Hspa1* expression within the studied rat brain areas.

### 2.2. Short-Term Selective REMS Deprivation Suppresses Deep SWS and Induces REMS Rebound but Does Not Affect Hspa1 Expression

We used selective REMS deprivation (REMSD) to determine the dependence of *Hspa1* upregulation, occurring during the daily REMS maximum, on the preceding deep SWS maximum. The deprivation started at ZT00, the lights-on period. Thus, the recovery period after the deprivation fell on the second half of the lights-on period (ZT06-12), when REMS is predominant, and the highest REMS rebound coincided with the natural daily maximum REMS, as was determined in experiment 1, described in the previous section.

Polysomnographic data indicated that the most prominent loss of REMS occurs over the first 2 h of REMSD. Animals showed 71.7% of REM sleep loss with only 2.4 ± 0.79% of residual REM sleep (Table 1). The residual REMS consisted of short episodes as the operator required about 10 s to identify and turn on the shaker to interrupt REMS. While the number of REM sleep episodes did not change significantly during the first 2 h of the deprivation compared to the control group, REM episodes became eight times shorter (Table 1). Over the next 4 h of the deprivation, REMS propensity steadily rose. This manifested in the progressive growth of residual REMS TT, which resulted from an increase in the number of attempts to enter REMS, while full-length REMS episodes were absent (Table 1). Therefore, the short-term REMSD challenged the homeostatic processes governing REMS, as residual REMS and shorter REMS episodes indicate an increase in REMS pressure during the deprivation in rats.

**Figure 5:**
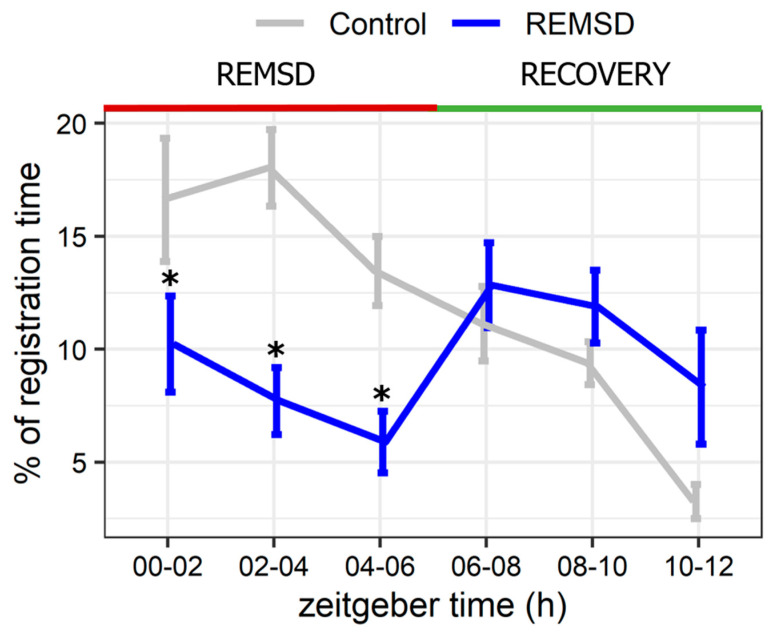
The reduction of deep slow-wave sleep during acute REM sleep deprivation (lights-on period, ZT00-06, REMSD) in Wistar rats without its subsequent rebound in the recovery period (ZT06-12, REC). Control—baseline recordings (*n* = 5 rats). REMSD—6 h selective REM sleep deprivation using an orbital shaker (*n* = 5 rats). *Y*-axis: % of deep SWS stage of total registration time. *X*-axis: zeitgeber time, h. Values are shown as mean ± s.e.m. *—*p* < 0.05 vs. control group, t test.

The last 4 h of REMSD altered SWS. Despite the increased attempts to enter SWS, its average episode duration was shortened twice, which resulted in a decrease in SWS TT by 22% compared to the control (Table 1). Accordingly, animals spent less time in the deep SWS stage throughout REMSD (Figure 5). The changes in SWS parameters indicated sleep fragmentation and the suppression of deep SWS during short-term REMSD in rats.

The loss of deep SWS was accompanied by a compensatory increase in drowsiness state. The quantity of drowsiness grew progressively, reaching up to one-third of total registration time due to more frequent attempts to enter this state after REMS interruption (Table 1). Wakefulness TT did not change during 6 h of REMSD because its episodes shortened, while the number of episodes increased two-fold compared to the baseline (Table 1).

Over 6 h of the recovery period following REMSD (ZT06-12), rats showed increased REMS quantity (60.2 ± 3.2 min in Recovery versus 47.8 ± 3.3 min at the baseline). Animals entered REMS in 171 ± 34.8 s on average once REMSD was finished. The most profound REMS rebound was observed during the first 2 h of the recovery period when REMS increased by 31.8% compared to the baseline (Table 1). This increase resulted from the tendency to prolonged REMS episodes with the number of episodes unchanged (Table 1). Then, REMS gradually decreased and reached control levels by the start of the lights-on period. REMS rebound was not accompanied by any significant changes in other vigilance states’ temporal characteristics as TT of wakefulness, drowsiness and SWS stayed close to their respective baseline levels (Table 1). Thus, despite the decreased quality and quantity of SWS during REMSD, the 6 h deprivation did not reach the critical point that would induce an SWS rebound in rats. 

Overall, short-term selective REMSD is effective for the acute induction of REM sleep rebound without any additional increase in SWS or drowsiness during the recovery period. However, it significantly suppressed the deep SWS as it caused a two-fold reduction in deep SWS TT without its subsequent rebound in the recovery period. Suppression of the deep SWS stage during REMSD seemed to result from accumulated REM sleep propensity in response to its deficiency. This manifested in the progressive growth of short residual REM sleep episodes due to an increased number of attempts to enter REM sleep after 2 h of deprivation, which has also been described earlier [40]. Increased frequency of REM sleep transitions prevented deep SWS from developing and hence led to SWS fragmentation. Such a relationship between REM sleep and SWS during sleep deprivation is normally observed in rodents (see, for example [41,42,43]). Nevertheless, during the recovery, we observed no statistically significant SWS rebound and light SWS was mostly preserved during REMSD and partially compensated by increased drowsiness. Overall, SWS reduction during REMSD was not physiologically significant to induce a notable SWS rebound. Hence, the anticipated changes in the *Hspa1* expression might be attributed either to REMS only or to the changes in proportion between REMS and deep SWS.

**Figure 6:**
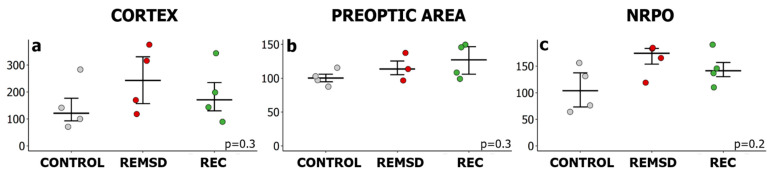
Acute selective REM sleep deprivation (REMSD) does not induce *Hspa1* upregulation during the highest rebound of REM sleep in the recovery period (REC). Relative *Hspa1* expression level in (**a**) the sensorimotor cortex, (**b**) the preoptic area of the hypothalamus and (**c**) the nucleus reticularis pontis oralis (NRPO) under REMSD conditions. The values were calculated using the 2^−ΔΔCt^ method, where the control group of undisturbed animals was used as the reference, ZT06 (CONTROL, grey). REMSD, red—rats were sacrificed after REMSD, ZT06; REC, green—rats were sacrificed after an episode of REMS 2 h into the recovery period, ZT08. Values are shown as median ± 75/25 percentile. Each dot represents an independent sample (animal). *p* values were calculated using the Kruskal–Wallis H test.

**Figure 7:**
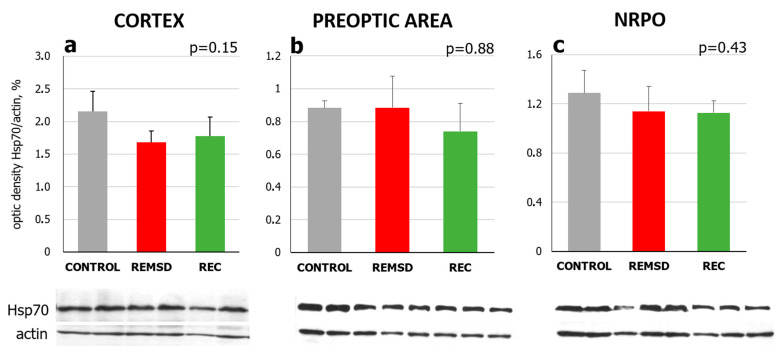
Hsp70 protein levels in (**a**) the sensorimotor cortex, (**b**) the preoptic area of the hypothalamus and (**c**) the nucleus reticularis pontis oralis (NRPO) after 6 h selective REM sleep deprivation (REMSD) and during the highest REM sleep rebound in the recovery period (REC). CONTROL, grey—undisturbed animals, ZT06. REMSD, red—rats were sacrificed after REMSD, ZT06; REC, green—rats were sacrificed after an episode of REMS 2 h into the recovery period, ZT08. Values are shown as mean ± s.e.m, *n* = 3 for each group. *p* values were calculated using the Kruskal–Wallis H test. Representative immunoblots are shown under the respective graphs.

To assess whether REMSD affects inducible chaperone Hsp70 expression in the brain, Hsp70 mRNA and protein levels were measured right after the end of REMSD and 2 h into the recovery period where the highest REMS amount was observed. Undisturbed animals were used as a control group. Real-time qPCR analysis did not identify an effect of REMSD on *Hspa1* expression within the sensorimotor cortex, the POA or the NRPO. The Kruskal–Wallis test did not show any significant difference between Control, REMSD and Recovery groups for mRNA (Figure 6, H(2, N = 12) = 2.19, *p* = 0.3; H(2, N = 11) = 2.23 *p* = 0.3; H(2, N = 12) = 3.23, *p* = 0.2, according to the brain structure) or protein level (H(3, N = 12) = 0.74, *p* = 0.86; H(3, N = 12) = 1.05, *p* = 0.79; H(3, N = 12) = 0.44; *p* = 0.93, according to the brain structure, Figure 7). Therefore, increased REMS, induced after short-term REMSD, seemed not to trigger Hsp70 expression in the studied areas of the rat brain, despite significant REMS rebound during the recovery period. This may be attributed to alterations in the natural deep SWS/REMS ratio induced by the selective REMSD, as further discussed in Section 3.2.

## 3. Discussion

### 3.1. Hspa1 Expression Is Upregulated in the NRPO during the Natural Daily Maximum of REM Sleep in Rats

To the best of our knowledge, the current study is the first to evaluate *Hspa1* expression in the brain during REMS and its association with the preceding deep SWS. The results indicate that, during the second half of the inactive (lights-on) period, the four-fold increase in REMS is accompanied by the three-fold increase in *Hspa1* expression in the NRPO compared to the wakefulness. The changes are associated with the REMS state, and not with the circadian rhythm since *Hspa1* expression is not upregulated in animals sacrificed at the same time point after a wakefulness episode.

However, the Hsp70 protein level within the NRPO seems to be delayed and does not change significantly despite the *Hspa1* upregulation. This could mean Hsp70 protein accumulation within the NRPO is likely to appear later than *Hspa1* upregulation or, at the given moment, has not reached the threshold required to detect significant changes by immunoblotting. Other studies yield similar findings, demonstrating a delayed increase in Hsp70 levels when *Hspa1* expression is triggered by heat shock stress and pharmacological agents. For example, the increase in Hsp70 level in mammalian cells that could be detected by immunoblotting occurs 3–6 h after heat shock preconditioning in vitro [44,45] and 4 h after geldanamycin treatment [46] or a day after administering an Hsp70 inducer U133 [47] in vivo, although transcriptional activation is detectable as early as in the first hour after the heat shock [45,48].

The NRPO is a part of central REMS regulation, and the upregulation of *Hspa1* during REMS could be associated with the enhancement of neuronal activity patterns and EEG activation with REMS onset. The NRPO is the pontine zone, cholinergic stimulation of which causes a rapid transition to REMS showing all the characteristic polysomnographic features (activation of the cerebral cortex, theta rhythm, muscle atony, ponto-geniculo-occipital waves, rapid eye movements) and lesions in this area prevent the development of REMS [49,50]. The key role of the NRPO in the REMS generation is provided by its connections with other brain structures responsible for polysomnographic features of REMS or involved in the regulation of sleep and wakefulness [50]. Along with REMS-on neurons, the NRPO contains other types of neurons which regulate wakefulness and SWS [51,52] and are likely to contribute to *Hspa1* expression variability “diluting” the changes in mRNA content produced by a small subgroup of cells, which we observe in this study. The heterogeneous nature of NRPO is illustrated by Deurveilher et al., who have shown that only about 12.5% of NRPO neurons induce REM sleep [51]. It is also supported by the study that identified a local population of cholinergic neurons with REM-specific discharge patterns in the pontine area, using optogenetics and juxtacellular recordings [53]. 

Thus, REMS during the second half of the inactive period creates conditions adequate for the expression of inducible Hsp70 in the NRPO. However, it is currently unclear what mediates the induction of Hsp70 during REM sleep. Increased protein synthesis, which apparently occurs in the preceding deep SWS, may also contribute. Hsp70 expression during REMS possibly serves as a restorative function, promoting chaperoning of misfolded proteins that accumulated during SWS at the beginning of the inactive period. This aligns with the notion that REMS serves the need created by the SWS and not by wakefulness [10,42]. 

We found no sleep-dependent changes in Hsp70 mRNA levels within the sensorimotor cortex or the POA in rats. This may be attributed to regional heterogeneity in the rate of protein synthesis across brain structures during natural deep SWS [18]. The need for Hsp70 induction in natural REMS may differ in different brain regions as well. Ambiguous results have also been obtained using total sleep deprivation in rodents. No induction of Hsp70 is found in the POA and sensorimotor cortex in mice [23]. However, later works [24] have shown increased Hsp70 amounts in the rat cortex after 8 h total sleep deprivation. An important result is described by Mackiewicz et al. [16], where an increase in the expression of *Hspa1* (Hsp70), *Hspa5* (Grp78/BiP), and other chaperones is shown in the mouse motor cortex, but only *Hspa5* is found to be induced in the hypothalamus. Such differences may arise from variations in the sleep deprivation method and/or in the sampling of different cortex areas, as well as from the species-specific features of the unfolded protein response.

### 3.2. Natural SWS-REMS Relation Should Be Preserved for the Hspa1 Upregulation to Occur

None of the existing hypotheses covering sleep function explain why SWS and REMS alternate in an ultradian sleep cycle and why REMS-enriched cycles follow an increase in SWS in the daily sleep cycle. Experimental data support the idea that both sleep stages are involved in neuroplasticity and biosynthesis of proteins and other macromolecules, but it is still unclear whether SWS and REMS serve the same plasticity processes [10]. Experimental separation of SWS and REMS function is difficult, partly because it is almost impossible to study these two vigilance states independently, and because selective REMS deprivation inevitably disrupts SWS temporal characteristics and SWA. Moreover, model rodents have extremely short sleep cycles, and it is challenging to measure biochemical and molecular parameters during each sleep stage separately [10]. 

This problem may be partially solved if REMS is seen as serving the need arising from SWS, as was proposed by Benington and Heller back in 1994 [42]. They argue that there is a homeostatic relationship between SWS and REMS; REMS propensity grows during the preceding SWS episodes until the pressure leads to a transition to REMS, which resolves this need. The present study supports this view.

In the natural sleep–wake cycle (control conditions, or “circadian model”), *Hspa1* expression in the NRPO shows a three-fold increase during the daily REMS maximum that occurs 5–7 h after the SWS maximum (Figure 10). The fact that SWS peaks shortly after the sleep onset is known for many warm-blooded animals [1,54]. Deep SWS also dominates over REMS in the recovery period after total sleep deprivation in rats when SWS rebound precedes and even suppresses REMS rebound [43,55]. The delayed increase in Hsp70 expression may reflect molecular mechanisms contributing to this temporal integration of SWS and REMS. The latency period may result from the deep SWS-dependent accumulation of misfolded proteins, which stimulate chaperone expression [21,56]. Up to 30% of nascent protein molecules are misfolded and undergo proteasomal degradation [20], and a substantial fraction of molecules (15 to 30% of mammalian proteomes) is not folded properly [57]. Such polypeptides are dysfunctional, sometimes toxic to the cell, are prone to spontaneous aggregation [31], and can accumulate during deep SWS when the rate of protein synthesis increases. Increased expression of the major chaperone Hspa70 in NRPO during REMS can prevent the synthesis of abnormal proteins, thereby contributing to their repair and maintaining cellular proteostasis and proteome functionality. Along with chaperones, the maintenance of protein balance is facilitated by the activation of the glymphatic system, which cleans the brain of toxic metabolites and abnormal proteins during deep SWS [58].

In the selective REMS deprivation model, REMS rebound exceeds the natural daily maximum. However, Hsp70 expression within the studied structures does not change. Hence, the main condition for *Hspa1* upregulation, which is a maximum of REMS, is met in the REMSD model, but this is not enough.

**Figure 8:**
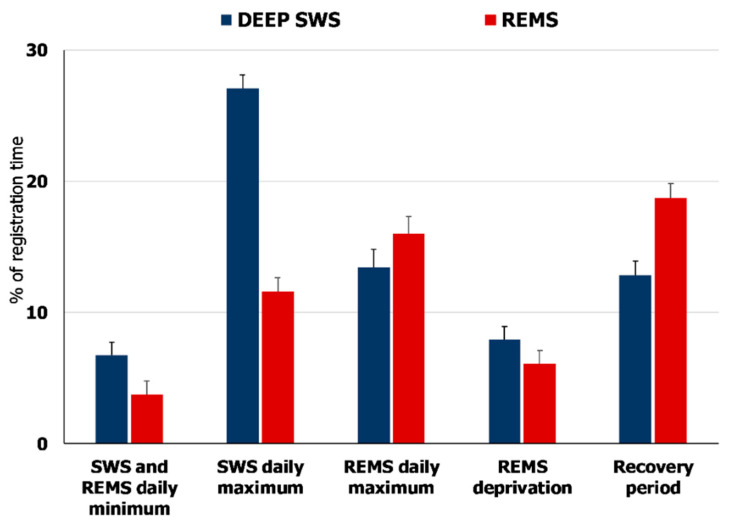
Total time of deep slow-wave sleep (DEEP SWS) and REM sleep (REMS) over the natural daily sleep–wake cycle (daily minimum and maximum of both states), during 6 h selective REM sleep deprivation (REMS deprivation) and the recovery period after the deprivation. Values are shown as mean ± s.e.m.

As another condition for Hsp70 expression during REMS, we consider the preservation of the deep SWS maximum at the beginning of the inactive period. In the “circadian model”, deep SWS peaks 5–7 h before the daily REMS maximum, and deep SWS total time is almost 3.5 times higher than in the REMSD model (Figure 8, comparison between the second and fourth pairs, blue bars). The dramatic decrease in deep SWS sleep during REMSD may presumably imply the following: there are fewer sleep cycles enriched with deep SWS; SWS-related protein synthesis [18] is thus suppressed, and the accumulation of misfolded proteins is likely to be prevented. Hence, it eliminates the need for elevated chaperone levels to protect cells against toxic protein molecules and *Hspa1* is not upregulated in the rat brain. Our assumption is partly supported by evidence of a general slight decrease in protein synthesis in the cerebellum, telencephalon and crude subcellular fractions of the brainstem of adult rats selectively deprived of paradoxical sleep [59,60].

The disruption of the natural temporal balance between SWS and REMS can be thus interpreted as a condition, unfavourable for *Hspa1* expression. The variation in *Hspa1* expression levels between animals may result from individual differences in deep SWS timing and SWS-REMS balance both in the “circadian” and REMSD models. Craig Heller [10] has repeatedly advised assessing the effects of REMSD cautiously, and we fully support this point of view. Still, this form of sleep deprivation allows for studying diverging changes of both sleep stages (preserved or even increased REMS but decreased deep SWS) which are separated temporally yet seem to be united by the common restorative function concerning neuronal proteostasis.

Our REMSD protocol has limitations because it requires constant monitoring by an operator experienced in sleep scoring; thus, it is mostly suitable for short-term deprivations on 1–2 animals simultaneously. Another potential drawback is that even short-term REMSD disrupts SWS quality due to an increase in the frequency of attempts to enter REMS. It is thus hard to isolate change attributed to reduced REMS specifically. However, we report no rebound of deep SWS over the 6 h recovery period after REMSD, which suggests that SWS disruption has a much less physiological significance compared to REMS loss during the deprivation. However, our experimental design, as well as other methods such as the use of transgenic animals, epigenetic and post-translational modifications, proteomic and whole-genome studies, is inevitably limited since sleep stages cannot be isolated as independent variables. Almost all physiological parameters (e.g., body temperature, hormonal background, respiratory rate, brain metabolism, feeding, and reproductive behaviour) change during sleep. Hence, it is virtually impossible to determine, for example, whether the change in the expression of a particular molecule that correlates with sleep or sleep deprivation is a direct consequence of sleep or some other concurrent physiological process.

The study is particularly limited by the assessment of the expression of a single target gene using real-time PCR; therefore, the overall effect may not reflect differences between neuronal populations and does not account for other chaperones involved in sleep regulation, such as Hsc70 or Grp78. Nevertheless, our findings provide a basis for implementing other techniques, including in situ hybridisation, to achieve cellular-level precision and to identify the neurons expressing Hsp70 when stimulated by REMS, as well as to study age- and sex-related features of chaperones’ involvement in sleep regulation. Further research is also required to identify how different sleep-regulating neuronal populations contribute to the changes in Hsp70 and other chaperones’ expression. Determining the spectrum of molecular differences in chaperone expression in the brain during REMS could guide further studies to validate the role of REMS in neuroprotection and recovery.

Future research can be directed to the investigation of the role of heat shock proteins in other essential functions of sleep, e.g., neuronal and behavioural development and memory formation, since the HSF1 transcription factor that induces Hsp70 expression plays an important role in hippocampal neurogenesis [61] and synaptic fidelity and function [62]. Understanding the role of chaperones in molecular mechanisms maintaining proteostasis and sleep–wake cycle regulation could be applied to the development of new therapeutic approaches targeting sleep and behaviour disorders, especially those associated with ageing and neurodegenerative diseases that disrupt both chaperone expression and deep SWS [47,63,64,65].

## 4. Materials and Methods

### 4.1. Animals

Adult 6–7-month-old male Wistar rats (m = 350–400 g) were individually housed in cages placed in the chamber with the controlled conditions: 12:12 h light–dark cycle with the lights-on period 7:00 (zeitgeber time, ZT00)–19:00 (ZT12), temperature 22 ± 1 °C and food and water available ad libitum. The experiments were conducted under the requirements of the European Community Council Directive 86/609/EEC and those of the Sechenov Institute of Evolutionary Physiology and Biochemistry of the Russian Academy of Sciences on the treatment of laboratory animals.

### 4.2. Surgical Procedure

Animals were operated under deep anaesthesia with Zoletil-100 (100 mg/kg i.m., Virbac, Carros, France). A pair of stainless-steel bipolar EEG electrodes was placed supradurally over each hemisphere using a stereotaxic device (2.0 mm lateral to the midline and +2.0 or −5 mm from bregma for the frontal and parietal electrodes, respectively). One electrooculogram (EOG) electrode was inserted into the orbital fossa; the other one was inserted in the nasal bone as the reference electrode. All electrodes were fixed to the skull with dental cement. Wire electrodes for EMG were implanted in dorsal neck muscles. The electrodes were connected to a sterile multi-channel telemetric device, type 4-ET (Data Sciences International Inc., New Brighton, MN, USA). The telemetric device was implanted subcutaneously according to the manufacturer’s guide. The rats were given at least 10–14 days to fully recover before starting EEG recordings for further analysis. 

### 4.3. Sleep Recording and Selective REM Sleep Deprivation (REMSD) Procedure 

Polysomnographic data were acquired with the telemetric recording system (Data Sciences International, USA). The 4-ET transmitters digitally monitored EEG, EOG, EMG, and subcutaneous temperature in non-restricted animals kept in their home cages. All bioelectrical signals were transmitted to a receiver RPC-2, converted analog to digital using the Dataquest A.R.T. software (Data Sciences International, Inc., New Brighton, MN, USA).

To minimise physical activity and stress, we designed the REMS interruption method by placing a cage on an orbital shaker SkyLine (ELMI SIA, Riga, Latvia). Our REMSD method was similar to the one described in the recent article [66] where REMSD was achieved through the induction of cage floor movements when REMS was detected. The authors showed that even 48 h deprivation did not induce blood corticosterone elevation; hence, deprivation with a shaker was also not likely to cause any significant stress.

REMSD was performed for 6 h (ZT00-06, lights-on period), and sleep stages were monitored remotely online by a trained operator. When the first signs of REMS appeared (theta-rhythm on EEG, loss of muscle tone on EMG, REM on EOG), an experimenter turned on the platform quickly, such that the animal returned to the SWS or brief wakefulness. Once the deprivation ended, rats readily fell asleep with the latency period until the first REMS being 171 ± 34.8 s, which suggests a minimal stress level compared to the platform-over-water method, which elicits a 30 min period of insomnia [67]. REMS rebound was comparable to the results of gentle handling [68]. 

### 4.4. Sleep–Wake Cycle Scoring and Analysis

Sleep recordings were scored offline by visual inspection, aided by custom Sleep Pro software developed in the MATLAB environment (The Math Works, Inc., Natick, MA, USA). The software filtered EEG signal to 0.5–100 Hz band, and EMG signal—to 10–100 Hz band; ADC sampling frequency—to 250 Hz. The episodes of each vigilance state were scored using a 10 s sliding time window. The states with a low-voltage, high-frequency, desynchronised EEG activity and sustained elevated neck muscle tone were identified as wakefulness. A high-voltage EEG with dominant delta frequency (0.8–4 Hz), associated with decreased muscle tone and no phasic muscular activity, was defined as SWS. REMS was identified by a decrease in the EEG amplitude and a prominent theta rhythm (5–8 Hz), combined with the muscle atonia at the EMG. The transition state with mixed characteristics of both wakefulness and sleep (spindles; no predominance of delta or theta frequencies; no phasic movements) was assumed as drowsiness. For each vigilance state, corresponding light/dark phase mean values of total registration time, number, and duration of episodes were analysed. 

Spectral analysis was run for each 4 s recording segment using Fast Furrier Transform. Slow-wave activity (SWA) was measured during SWS and calculated as the ratio of EEG power spectrum in delta-range (0.8–4 Hz), for each recording hour to 24 h EEG power spectrum in 0.8–50 Hz range. Deep SWS was calculated for each animal as the amount of SWS with the highest SWA prevalence, which is the state that typically comprises 30% of total SWS time during the day under control conditions.

### 4.5. Experimental Procedure 

**Figure 9:**
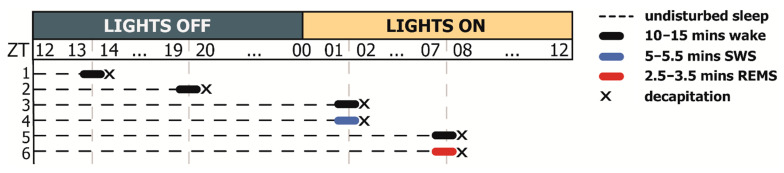
Experiment 1 scheme. To access *Hspa1* expression across the daily sleep–wake cycle and compare it with the polysomnographic data, six groups of rats were used (*n* = 6 in each group) according to the time of decapitation and the preceding vigilance state, where zeitgeber time (ZT) 00-12 is the lights-on period: (1) ZT13-14, wake; (2) ZT19-20, wake; (3) ZT01-02, wake; (4) ZT01-02, SWS; (5) ZT07-08, wake; (6) ZT07-08, REMS. Following real-time sleep recording, non-anaesthetized rats were immediately sacrificed after 10 min of wake, or 5–5.5 min of SWS, or 2.5–3.5 min of REMS.

Experiment 1. To study *Hspa1* expression during the natural sleep–wake cycle, rats (*n* = 10) were implanted with 4-ET telemetric modules (DSI, USA). The mean of 3 control 24 h sleep recordings was calculated for each animal, and the control group was used to identify the periods of maximal and minimal percentages of vigilance states. To access *Hspa1* expression across the daily sleep–wake cycle and compare it with the polysomnographic data, six groups (*n* = 6 each) of rats were used according to the time of decapitation and the preceding vigilance state (Figure 9). Following real-time sleep recording, non-anesthetised rats were immediately sacrificed after 10 min of wakefulness, or 5–5.5 min of SWS, or 2.5–3.5 min of REMS.

**Figure 10:**
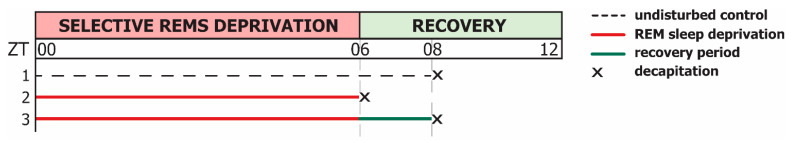
Experiment 2 scheme. Twenty-four-hour baseline recording was followed by 6 h selective REM sleep deprivation (REMSD, ZT00-06, lights on) and a 6 h recovery period (ZT06-12, lights on) for each animal. Three groups of rats were used (*n* = 4 in each group) for *Hspa1* expression analysis under REMSD conditions: (1) undisturbed control, ZT06; (2) REMS-deprived rats immediately after REMSD, ZT06; (3) REMS-deprived rats 2 h into the rebound period, ZT08.

Experiment 2. In the short-term selective REM sleep deprivation (REMSD) series, rats (*n* = 8) were implanted with 4-ET telemetric modules (Data Sciences International, Inc., New Brighton, MN, USA). Twenty-four-hour baseline recording was followed by 6 h REMSD (ZT00-06, lights on) and 6 h (ZT06-12, lights-on) of the recovery period for each animal. Three groups of rats were used (*n* = 4 each) for *Hspa1* expression analysis (Figure 10). 

For each rat in both experiments, the sensorimotor cortex, the preoptic area of the hypothalamus (POA), and the nucleus reticularis pontis oralis (NRPO), dissected according to the Wistar rat brain atlas [69], were randomly distributed for further Western blot and qPCR analysis.

Our REMSD protocol has limitations because it requires constant monitoring by an operator experienced in sleep scoring; thus, it is mostly suitable for short-term deprivations on 1–2 animals simultaneously. The study is also limited by the assessment of gene expression using real-time PCR; therefore, the overall effect may not reflect differences between neuronal populations.

### 4.6. Real-Time PCR and Western Blotting

Brain tissue samples were fixed either in IntactRNA solution (Evrogen, Moscow, Russia) for the following qPCR analysis, or in LowRIPA buffer for Western blot analysis, and were stored at −80 °C until use. Extraction of total RNA was performed using the phenol-chloroform method (ExtractRNA solution by Evrogen, Moscow, Russia) according to the manufacturer’s instruction. Total RNA was quantified with the Nanophotometer (Implen GmbH, München, Germany) by measuring absorbance at 260, 280, and 320 nm. Reverse transcription was run with an oligodT primer and the MMLV RT kit (Evrogen, Moscow, Russia) according to the manufacturer’s instructions.

Gene-specific primers were designed using PrimerBLAST software (NCBI-NIH, Bethesda, MD, USA) to span an exon-intron boundary and exclude amplification of genomic DNA wherever possible. The genes studied and primers used were: (1) *B2m* (NM_012512.2): F-TGGGGAGTTTTCTGAATGGC, R-TCGGTGACCGTGATCTTTC; (2) *Gapdh* (NM_017008.4): F-TCTCTGCTCCTCCCTGTTCTA, R-GGCCAAATCCGTTCACACC; (3) *Hprt* (NM_012583.2): F-CTTCCTCCTCAGACCGCTTT, R-TCATCACTAATCACGACGCTG; (4) *Hspa1* (NM_031971.2, NM_212504.1): F-GACACCAGATTCCTCCTCTTAATC, R-CTCTCTGCTTCTGGAAGGTTG. *Gapdh*, *B2m*, and *Hprt* were chosen as internal controls for *Hspa1* expression normalisation. These house-keeping genes have stable expression levels over the normal sleep–wake cycle and after sleep deprivation [70,71]. Real-time qPCR was run in quadruplicates (10 ng per 25 µL well, 0.3 µM forward and reverse primer) on ABI 7500 detection system (Applied Biosystems, Thermo Fisher Scientific, Waltham, MA, USA) using qPCRmix-HS LowRox SYBR Green solution (Evrogen, Moscow, Russia). qPCR conditions comprised a 5 min preincubation at 95 °C, followed by 40 cycles of 15 s at 59 °C and 30 s at 72 °C (fluorescence detection step). Each run was followed by a melting curve protocol to check for product specificity. After amplification, the cycle threshold (Ct) was drawn from ABI 7500 v.2.3 software (Applied Biosystems, Thermo Fisher Scientific, Waltham, MA, USA) and analysed using the comparative ΔΔCt method with *Hspa1* as a gene of interest and *Gapdh*, *B2m*, and *Hprt* as reference genes. 

For Western blot analysis, total protein was extracted from brain tissue samples with LowRIPA buffer. Samples were separated by 10% SDS-polyacrylamide gel electrophoresis and electroblotted onto PVDF membranes. The membranes were stained with primary antibodies against inducible Hsp70 (rabbit, 1:2000, Sigma-Aldrich, Merck KGaA, Darmstadt, Germany) and GAPDH or β-actin (mouse or goat, 1:2000, Abcam, UK) as loading controls and subsequently with secondary antibodies conjugated with horseradish peroxidase (1:10,000; Abcam, Cambridge, UK). The antigen/antibody complex was detected with an enhanced chemiluminescence method, using ECL solution (Invitrogen, Thermo Fisher Scientific, Waltham, MA, USA). Densitometric analysis was performed in open-source ImageJ 1.8 software. 

### 4.7. Statistical Analysis

The data were analysed using Statistica 8.0 software (Stat Soft Inc., Tulsa, OK, USA). The t test for dependent samples was applied to assess the difference in sleep–wake cycle parameters between the control and REM sleep deprivation groups. A non-parametric Kruskal–Wallis test for multiple comparisons, followed by a Mann–Whitney post hoc test (where applicable), was run for Hsp70 mRNA and protein level comparison across experimental conditions. A statistical difference was considered to be significant when *p* level < 0.05 for all statistical procedures.

## 5. Conclusions

The results show for the first time that under natural physiological conditions, increased REMS may induce the expression of the *Hspa1* gene, encoding the Hsp70 chaperone, in the brainstem. This REMS function seems to be dependent on the preceding increase in deep SWS. The data obtained puts forward a new perspective: increased *Hspa1* expression during REMS maximum in the second half of the inactive period may serve as a neuroprotective response to the negative consequences of intensified protein synthesis occurring in deep SWS during the first half of the inactive period. This molecular mechanism can potentially contribute to eliminating the accumulated conformational changes in protein molecules, thus “protecting” a key restorative function of sleep and mediating the integration of the two states (SWS and REMS) in the daily sleep cycle. 

## Figures and Tables

**Table 1:** Temporal characteristics of the sleep–wake cycle during and after short-term selective REM sleep deprivation (REMSD) in rats.

State and Time, ZT		Total Time ^1^, % of Registration Time		Episode Duration ^1^, s		Number of Episodes ^1^	
		Control ^2^	REMSD ^2^	Control	REMSD	Control	REMSD
Wakefulness	00-02	35.3 ± 4.92	34.5 ± 4.73	244 ± 34.7	124 ± 27.2 *	11 ± 0.9	22 ± 2.1 *
	02-04	19.4 ± 4.08	25.8 ± 5.05	115 ± 21.6	78 ± 18.6	14 ± 2.2	27 ± 4.6 *
	04-06	29.5 ± 3.54	30.7 ± 4.33	174 ± 21.8	91 ± 17.1 *	14 ± 2.0	27 ± 4.0 *
	06-08	24.3 ± 4.25	19.9 ± 3.39	159 ± 40.8	131 ± 28.9	12 ± 1.4	11 ± 0.9
	08-10	22.7 ± 4.05	21.5 ± 3.86	150 ± 24.5	147 ± 41.6	10 ± 0.9	12 ± 1.8
	10-12	37.3 ± 5.46	32.8 ± 5.62	273 ± 49.7	249 ± 60.0	10 ± 1.5	11 ± 1.1
Drowsiness	00-02	16.1 ± 2.44	23.3 ± 2.89	41 ± 3.8	41 ± 3.3	28 ± 3.0	41 ± 4.7 *
	02-04	17.4 ± 2.10	28.2.2 ± 2.66 *	39 ± 2.2	35 ± 2.8	36 ± 5.2	61 ± 6.3 *
	04-06	18.6 ± 2.60	27.6 ± 3.21 *	44 ± 5.0	34 ± 2.7	32 ± 3.2	59 ± 4.4 *
	06-08	19.4 ± 1.82	17.3 ± 2.52	43 ± 2.9	40 ± 3.7	33 ± 2.8	30 ± 2.3
	08-10	20.1 ± 3.52	19.5 ± 3.30	42 ± 3.7	42 ± 4.7	32 ± 3.7	34 ± 3.9
	10-12	21.5 ± 4.35	20.0 ± 2.48	50 ± 4.8	44 ± 5.3	29 ± 3.1	34 ± 2.9
Slow-wave sleep	00-02	40.0 ± 3.19	39.7 ± 2.39	138 ± 14.8	101 ± 7.8 *	23 ± 2.9	30 ± 3.3
	02-04	50.4 ± 3.95	38.0 ± 1.85 *	155 ± 25.0	73 ± 7.0 *	26 ± 2.6	40 ± 4.3 *
	04-06	41.3 ± 2.72	33.4 ± 2.58 *	136 ± 15.1	64 ± 8.2 *	24 ± 2.0	40 ± 4.3 *
	06-08	41.7 ± 2.28	43.7 ± 2.67	125 ± 11.9	132 ± 11.5	26 ± 2.5	24 ± 1.8
	08-10	42.6 ± 2.84	41.6 ± 2.15	129 ± 13.9	126 ± 24.4	25 ± 2.8	27 ± 2.8
	10-12	30.3 ± 3.29	34.2 ± 3.80	107 ± 21.0	97 ± 8.4	22 ± 2.5	26 ± 2.8
REM sleep	00-02	8.5 ± 1.27	2.4 ± 0.79 *	128 ± 12.3	17 ± 2.0 *	5 ± 0.9	10 ± 3.0
	02-04	12.9 ± 1.21	8.0 ± 1.62 *	166 ± 14.5	18 ± 1.9 *	9 ± 4.1	32 ± 5.5 *
	04-06	10.6 ± 1.35	8.4 ± 1.58	145 ± 11.1	21 ± 5.3 *	8 ± 2.5	35 ± 6.7 *
	06-08	14.5 ± 1.51	19.1 ± 1.42 *	149 ± 13.3	169 ± 13.5	7 ± 0.7	8 ± 1.0
	08-10	14.6 ± 1.62	17.4 ± 1.33	157 ± 10.3	151 ± 14.7	7 ± 0.9	9 ± 0.8
	10-12	10.9 ± 1.80	13.0 ± 1.82	161 ± 15.2	172 ± 12.1	5 ± 1.8	6 ± 1.3

^1^—Values are shown as mean ± s.e.m. ^2^—Control (*n* = 8)—baseline recordings, no sleep deprivation; REMSD (*n* = 8)—6 h selective REM sleep deprivation (ZT00-06) and recovery period (ZT06-12). * indicates a statistically significant difference in temporal characteristics between REMSD and corresponding control, *p* < 0.05, *t* test for dependent samples.

## Data Availability

The data presented in this study are available on request from the corresponding author IVE.
